# Zebrafish larvae show negative phototaxis to near-infrared light

**DOI:** 10.1371/journal.pone.0207264

**Published:** 2018-11-28

**Authors:** Sarah Hartmann, Roland Vogt, Jan Kunze, Anna Rauschert, Klaus-Dieter Kuhnert, Josef Wanzenböck, Dunja K. Lamatsch, Klaudia Witte

**Affiliations:** 1 Research Group of Ecology and Behavioural Biology, Institute of Biology, Department of Chemistry-Biology, University of Siegen, Siegen, Germany; 2 Research Department for Limnology, Mondsee, University of Innsbruck, Mondsee, Austria; 3 Institute of Real-time Learning Systems, Department of Electrical Engineering and Computer Science, University of Siegen, Siegen, Germany; Institut Curie, FRANCE

## Abstract

Zebrafish larvae (*Danio rerio*) are among the most used model species to test biological effects of different substances in biomedical research, neuroscience and ecotoxicology. Most tests are based on changes in swimming activity of zebrafish larvae by using commercially available high-throughput screening systems. These systems record and analyse behaviour patterns using visible (VIS) and near-infrared (NIR) light sources, to simulate day (VIS) and night (NIR) phases, which allow continuous recording of the behaviour using a NIR sensitive camera. So far, however, the sensitivity of zebrafish larvae to NIR has never been tested experimentally, although being a critical piece of information for interpreting their behaviour under experimental conditions. Here, we investigated the swimming activity of 96 hpf (hours post fertilization) and 120 hpf zebrafish larvae under light sources of NIR at 860 nm and at 960 nm wavelength and under VIS light. A thermal source was simultaneously presented opposite to one of the light sources as control. We found that zebrafish larvae of both larval stages showed a clear negative phototactic response towards 860 nm NIR light and to VIS light, but not to 960 nm NIR light. Our results demonstrated that zebrafish larvae are able to perceive NIR at 860 nm, which is almost identical to the most commonly used light source in commercial screening systems (NIR at 850 nm) to create a dark environment. These tests, however, are not performed in the dark from the zebrafish´s point of view. We recommend testing sensitivity of the used test organism before assuming no interaction with the applied light source of commonly used biosensor test systems. Previous studies on biological effects of substances to zebrafish larvae should be interpreted with caution.

## Introduction

Fish in general are sensitive model species and have been frequently used in automated biological monitoring systems as biosensors [1]. The analysis of behavioural parameters such as swimming activity, respiration, shoaling behaviour or rheotactic behaviour are of particular interest [1–3]. Measuring sublethal effects as behavioural endpoints leads to a higher significance and, compared to mortality, a 10–100 times higher sensitivity can be achieved [4, 5].

During the last few decades the zebrafish (*Danio rerio)*, a small tropical freshwater fish, became one of the most used model species in the field of biomedical research, neuroscience and ecotoxicology [6–11]. Breeding and cultivating this fish species in the lab is cost-effective and embryo development is very fast [12]. Additionally, the complete genome of the zebrafish is known [13] and has broad homologies to other vertebrate species [14–16], and genes involved in behaviour are highly conserved between humans and zebrafish [9]. Thus, monitoring of zebrafish swimming activity serves as a sensitive and powerful tool for identifying toxic compounds in several fields of research [1, 3]. These behavioural measurements are highly economical and appropriate for e.g. ecotoxicological research [17]. They are mostly based on swimming activity of adult zebrafish and zebrafish larvae, which are monitored using video tracking systems and corresponding software to detect and quantify changes in locomotion pattern. So far, commercially available high-throughput tracking systems, such as the DanioVision from Noldus (Wageningen, Netherlands) or the Zebrabox from Viewpoint (Lyon, France) allow tracking of locomotion parameters of zebrafish larvae in multi-well plates. Many studies use light/dark transition tests to investigate possible effects of several substances (e.g. ethanol, cadmium, microplastic, neurotoxic drugs) on swimming activity of zebrafish larvae. The dark sections are lit with NIR light, so that the behaviour can still be recorded with an IR sensitive camera [3, 6, 16, 18–23]. The Zebrabox system e.g. uses an infrared light illumination at a wavelength of 850 nm to record the swimming activity under “dark” conditions [24]. So far, it has been assumed that zebrafish larvae cannot perceive NIR light and do not respond to it [3, 25, 26]. This assumption was based, first, on the anatomy of the vision system in adult zebrafish. Adult zebrafish have four different types of photoreceptors with specific visual sensitivity in the red spectrum (~570 nm), in the green spectrum (~480 nm), in the blue spectrum (~415 nm) and in the ultraviolet spectrum (~362 nm) [26–28]. Secondly, in an optokinetic experiment, zebrafish larvae showed no eye movement in response to rotating stripes that were illuminated with 750 nm NIR light [29]. It is known, however, that the visual system can change during ontogeny in fish [30, 31]. Nevertheless, it is precarious to extrapolate from the visual sensitivity in adult fish to the visual sensitivity in fish larvae within a species.

Recently, only a few researchers focused on near-infrared (NIR) sensitivity in fish, although the absorption spectra of light in natural aquatic ecosystems covers wavelengths higher than 700 nm [32, 33]. The sensitivity towards NIR illumination was reported in the common carp *Cyprinus carpio* and the Nile tilapia *Oreochromis niloticus* [34]. Both species showed a visual sensitivity towards the near infrared light at 865 nm, and a perception of even longer wavelengths (936 nm) was indicated in the common carp [34]. Due to the experimental set-up, the authors concluded that the NIR light was detected by the eyes and not by the pineal organ in both species, the common carp and the Nile tilapia [34]. In the cichlid *Pelvicachromis taeniatus* it could be shown that a direct illumination of the prey organism *Gammarus pulex* with NIR wavelengths between 780 and 920 nm led to stronger foraging responses compared to non-illuminated *G*. *pulex* [35] which indicates prey-detection through NIR sensitivity. Shcherbakov et al. (2013) [36] analysed the NIR detection under different light conditions as a parameter for spectral sensitivity in different fish species: the Mozambique tilapia (*Oreochromis mossambicus*) and the Nile tilapia (*O*. *nilticus*) showed a high sensitivity to wavelengths above 930 nm, and they found an upper threshold for the green swordtail (*Xiphophorus helleri*) at 825–845 nm. Furthermore, the authors investigated the response of adult zebrafish to NIR light and determined a threshold at 845–910 nm, as clear evidence for the perception of NIR by adult zebrafish [36]. The authors explained the sensitivity to NIR as an evolutionary adaptation to environmental conditions and suggested long wavelength sensitive cones as a potential candidate for NIR perception in fish [36].

Here, we investigated the behavioural reaction of zebrafish larvae at two larval stages, (96 hours post fertilization (hpf) and 120 hpf), to different wavelengths of NIR light (860 nm and 960 nm), to test for a phototactic response according to Jékley (2009) [37]. As Shcherbakov et al. 2013 [36] found that adult zebrafish are able to sense NIR light up to 910 nm, we hypothesized that zebrafish larvae might perceive a similar NIR light spectrum. We, therefore, conducted three experiments under specific light characteristics: NIR light with a spectral peak at 860 nm and 960 nm, and blue-white light as a visible light source (VIS, 440–700 nm). Our results showed for the first time that zebrafish larvae at both larval stages showed a clear negative phototactic response towards NIR light at 860 nm as well as towards VIS light, but not to NIR light at 960 nm wavelength. Our results are, therefore, highly relevant to all experiments using zebrafish larvae in standard testing procedures under NIR light conditions because most experimental devices use 850 nm light sources to simulate a dark environment. These tests, however, are not carried out in the dark from the zebrafish´s point of view.

## Material and methods

### Ethics statement

All experiments were non-invasive behavioural tests. The performed experiments were in line with the German Animal Welfare Act (Deutsches Tierschutzgesetz) and approved by the internal animal protection commissioner Dr. Urs Gießelmann, University of Siegen, Germany, and the national Veterinary Authority (Kreisveterinaeramt Siegen-Wittgenstein, Germany).

### Study species

We used *Danio rerio* of a wild-type zebrafish strain from West Aquarium GmbH (Bad Lauterberg, Germany). The *D*. *rerio* culture was kept in 112 L glass tanks (80 x 40 x 35 cm^3^) at a constant water temperature of 26 ± 1°C with a pH-value of 7–7.5 and a conductivity of 450 μS/cm under a light-dark cycle of 14:10 hours. Water was aerated and filtered continuously and partly exchanged (40%) once a week. Adult zebrafish were fed daily *ad libitum* with dry flake food (JBL GmbH & Co. KG, Germany), and with juvenile *Daphnia magna* three times a week to provide a source of environmental enrichment [38]. We placed a spawning tray (16.8 x 25.7 x 6.2 cm^3^) covered with artificial plants to stimulate egg laying into the home tank in the evening until the next morning, 2 h after onset of light. Collected eggs were rinsed with distilled water and placed into a petri dish (18.5 cm in diameter) for egg selection. Fertilized and healthy eggs were kept in 60 mL crystallisation dishes (60 x 35 mm^2^) for development until larvae were 96 hpf and 120 hpf and were cultured under the same conditions (water temperature: 26 ± 1°C; light-dark cycle 14:10) as adult zebrafish. Fish eggs were checked daily and dead and abnormal embryos were removed. Only normally developed and hatched larvae were used for testing.

#### Phototactic experiments and video tracking

**Fig 1:**
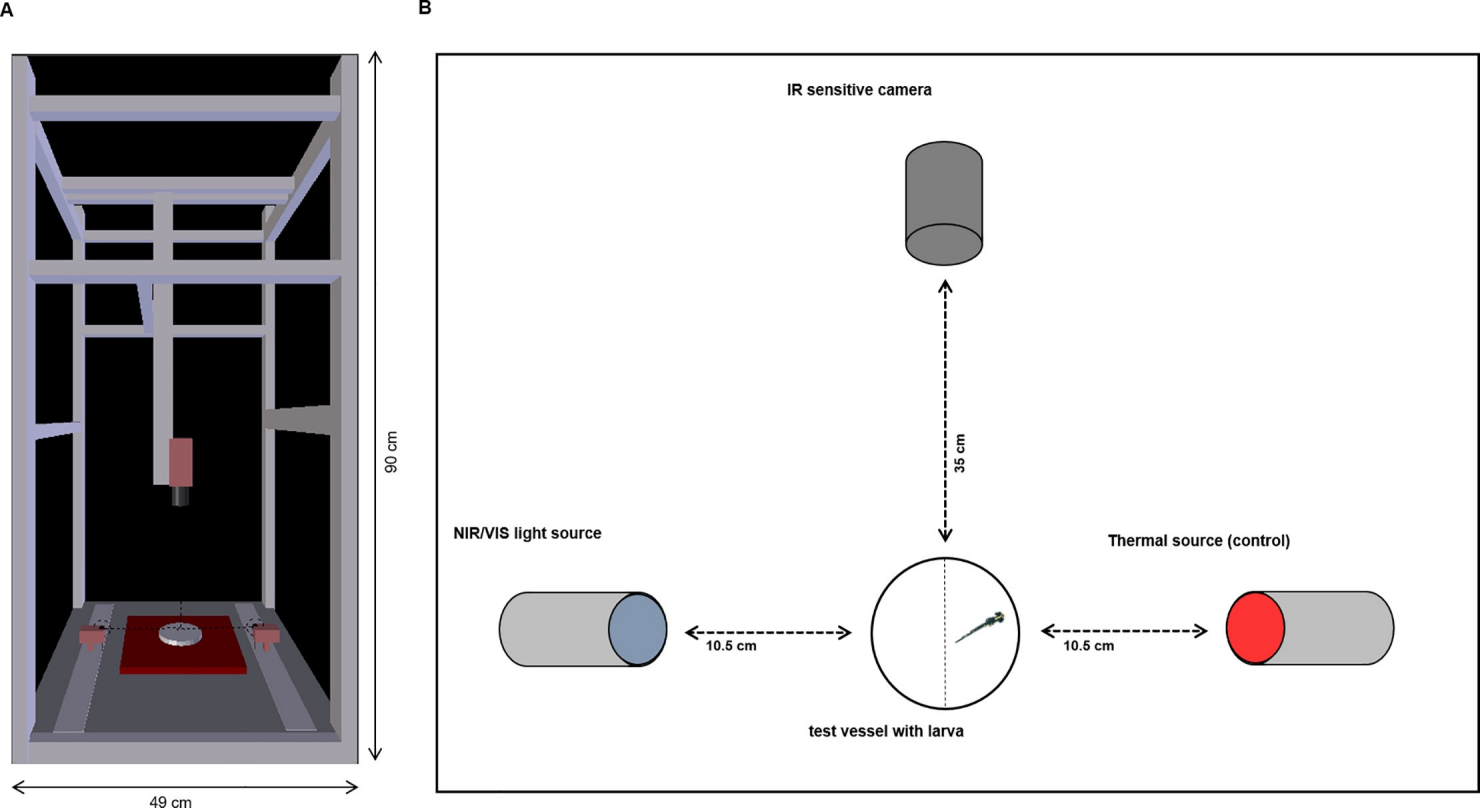
Scheme of the experimental set-up of the NIR light experiments. (A) The custom-built computer vision system shows the position of the test vessel in the middle of the set-up, the camera above the test vessel and the position of the light sources. The computer vision system was totally covered so that no light from the surrounding area could enter the system. (B) A detailed view of the set-up: For each experiment, one larva was placed into the test vessel. The IR sensitive camera was set 35 cm above the vessel, and both light sources were 10.5 cm away from the test vessel. A thermal source opposite to the NIR/VIS light served as a control. We switched the position of light and thermal sources after each trial to exclude side biases. The vessel was divided into 2 halves with an imaginary line, one half illuminated by the light source (VIS/NIR), one half radiated by the thermal source (control side) (drawing of zebrafish larva by Kimmel et al. 1996 [12]).

Phototactic experiments followed Shcherbakov et al. (2012) [39] with some modifications. All experiments were performed in a room with a constant temperature of 26 ± 1°C. We used a custom-built, light-isolated chamber coated with black PVC plates (49 x 90 x 45 cm^3^), to record movements of the zebrafish larvae in a petri dish (diameter: 35 mm), which served as the test vessel (Fig 1A), under specific light conditions for 5 minutes. Movement was recorded with an IR sensitive camera (Manta G-235C, Allied Vision, Stadtroda, Germany) with a frame rate of 35/s, fixed 35 cm above the test vessel. The camera was connected to a PC to control, start and manage the experimental settings. We performed three experiments with different light sources to test the sensitivity of the zebrafish larvae at an age of 96 hpf or of 120 hpf towards two different wavelengths of NIR light and VIS light. In each experiment a light source was provided simultaneously with a thermal source positioned opposite to the light source in 10.5 cm distance from that vessel (Fig 1A and 1B) to control for a thermal gradient within the test vessel. The thermal source was an 860 nm light source equipped with a 950-IR high-pass filter (IR-filter 950 nm, Delamax, Germany) that cut off all shorter wavelengths and additionally equipped with a UV-IR-cut filter (HD2130, Ningbo Haida Photo supplies Co., Ltd., Ningbo China) to cut off wavelengths between UV and NIR, so that light was entirely blocked (S1 Fig). To test for temperature differences, we measured the temperature of each light source 30 times after each 5 min test period with an IR thermometer at the position of the test vessel in 10.5 cm distance (IRT-350 IR thermometer, Base Tech, Hirschau, Germany). To test for a thermal gradient within the test vessel we measured the water temperature within the test vessel at five different positions (left and right side, centre, top and bottom) with a digital thermocouple (digital probe thermometer POCKET-DIGITEMP, TFA Dostmann GmbH & Co. KG, Wertheim-Reicholzheim, Germany) 5 min after onset of light, thus under same conditions as in trials. Furthermore, we measured the light intensity as irradiance [μWatt/cm^2^] and the radiated spectrum of all applied light sources and of the thermal source covered by used filters, respectively, with an AvaSpec-2048 spectrometer covering a range of 220–1100 nm (Avantes BV, Apdeldoorn, Netherlands, Europe) in 2 cm distance to the source.

We transferred one larva per trail to a 35 mm petri dish filled with 3 mL formulated water (294 mg/l CaCl_2_∙2 H_2_O, 123.3 mg/l MgSO_4_∙7 H_2_O, 63 mg/l NaHCO_3_, 5.5 mg/l KCl [40]). The vessel was placed under the IR sensitive camera (Fig 1B). Since the transfer of larvae was performed in light, we chose an acclimation time for each larva of 5 min within the device in darkness. Thereafter, light sources (VIS/NIR) and the thermal source were switched on and the swimming activity was recorded for 5 min. It was previously reported that the most stable activity period in zebrafish larvae ranges between 1:00 and 4:30 pm [19, 41]. Therefore, all experiments were carried out within this time period.

#### Phototactic experiments under VIS

In the first experiment, we tested the phototactic behaviour of zebrafish larvae under blue-white light (blue: LED type: 151053BS04500, Würth Eletronic, Waldenburg, Germany, spectral peak at 460 nm; white: LED type: LW340-A, Soeul Semiconducter Co., Ltd, Ansan, South Korea, spectral peak at 460 nm and 560 nm) which served as a visible light (VIS) source. A blue-white light source was used because zebrafish are able to see blue light [26, 27]. In order to ensure that the VIS light source did not reflect shorter or longer wavelengths, a UV-IR-cut filter (HD2130, Ningbo Haida Photo supplies Co., Ltd., Ningbo China) was attached to the device that absorbed ultraviolet and infrared wavelength (Table 1 and S1 Fig). The measured spectral range of the VIS light source was 420–680 nm, with a maximum absorption at 460 nm and a light intensity of 2.49 μWatt/cm^2^ (Table 1 and S1 Fig). Opposite to the VIS light source, we used the same thermal source as described above to provide thermal radiation only (S1 Fig). In total, we tested 30 larvae of each larval stage and exposed 15 larvae of each larval stage with VIS light from the right and 15 larvae with VIS light from the left side to exclude side biases.

**Table 1:** Spectral characteristics of the used light sources (VIS, NIR 860 nm and NIR 960 nm) for phototactic experiment.

Light spectrum	λ_(max)_ [nm]	measured spectral range [nm]	80% of the spectral range [nm]	Irradiance [μWatt/cm^2^]
**VIS**	460	420–680	455–645	2.49
**NIR 860 nm**	860	830–910	845–885	16.09
**NIR 960 nm**	960	890–1050	912–998	31.98

#### Phototactic experiments under NIR 860 nm light condition

In the second experiment, zebrafish larvae of 96 hpf or 120 hpf were illuminated with a light source with the peak illumination at 860 nm (LED type (850 nm): HE1-240AC, Harvatek Corp., Hsinchu City, Taiwan). The light source was covered with an IR filter (IR-filter 850 nm, Delamax, Germany) to eliminate visible light components (below 850 nm) and long wavelengths (above 910 nm), and to shift the maximum to the requested illumination (Table 1 and S1 Fig). Due to the applied filter, the measured spectral profile ranged from 830 to 910 nm with a maximum absorption at 860 nm and has a light intensity of 16.09 μWatt/cm^2^ (Table 1 and S1 Fig). Therefore, we refer this light source as 860 nm source throughout the text. A thermal source was placed opposite to the NIR light source (see above) to provide thermal radiation only (S1 Fig). We tested 30 larvae of each larval stage as described above.

#### Phototactic experiments under NIR 960 nm light condition

In the third experiment we used an NIR source with peak emission at 960 nm (LED type (940 nm): HE3-245AC, Harvatek Corp., Hsinchu City, Taiwan) which was equipped with an high-pass IR-filter at 950 nm (IR filter 950 nm, Delamax, Germany) to remove those visible light components with shorter wavelengths and to shift the maximum to the peak illumination of 960 nm (Table 1 and S1 Fig). Due to the applied filter measured spectral profile ranged from 890 to 1050 nm with a maximum absorption at 960 nm, with a light intensity of 31.98 μWatt/cm^2^ (Table 1 and S1 Fig). Therefore, we refer this light source as a 960 nm source throughout the text. We tested 30 larvae of each larval stage as described above.

### Behaviour analysis

Recordings of the zebrafish movements were analysed by a tracking software developed by the Institute of Real-Time Learning Systems, University of Siegen. The position of the larva (eye position) in the vessel was analysed every 2 s for 5 min and was performed manually through a marking between the eyes [39]. According to Shcherbakov et al. [36] the following default parameters were calculated: (I) swimming activity [%], with the maximum number of possible changes (= 150 within 300 s) in position (x- and y-value) in all analysed pictures set to 100% within a 5 min test period; (II) absolute allocation time [s] on each side of the test vessel; (III) relative allocation time [%] to illustrate the preferred area in the test vessel; (IV) preferred head orientation in relation to the corresponding light source measured as mean angle [°] (S2 Fig); and (V) head orientation as the length of the mean directional vector *R* as a scale of the concentration of data points around a circle [42]. The mean head orientation, as a directional parameter, is a good factor to measure the location of circular data and it is correlated to the direction of the length of the mean directional vector *R* of the data [42]. A value of *R* = 1 indicates, that all data points are located around the mean direction, a value near to 0 means evenly distributed data around the circle [42].

### Data analysis

Statistical analyses were carried out using R 3.2.4 for windows [43]. Before analysing allocation time, we defined an activity threshold excluding those larvae showing a swimming activity lower than 20% in the vessel during the recording. The threshold was set due to the known freezing behaviour of zebrafish larvae. We wanted to avoid a bias in our results due to freezing larvae on one side of the vessel. To test for differences in swimming activity [%] within a larval stage and between larvae of the two different larval stages under different light sources, respectively, we used a non-parametric Kruskal-Wallis rank sum test followed by the Wilcoxon rank sum test for unpaired samples. To test for differences in allocation time (control side vs. NIR/VIS light side) within one larval stage we used a Wilcoxon signed rank test. All P-values are two tailed and were adjusted with Bonferroni correction. A Rayleigh test was performed to test directional uniformity, to analyse the mean directional vector (*R*), and to assess directional preferences of larvae to a light source. Significance level was set to α = 0.05.

## Results

### Swimming activity under NIR and VIS light

**Fig 2:**
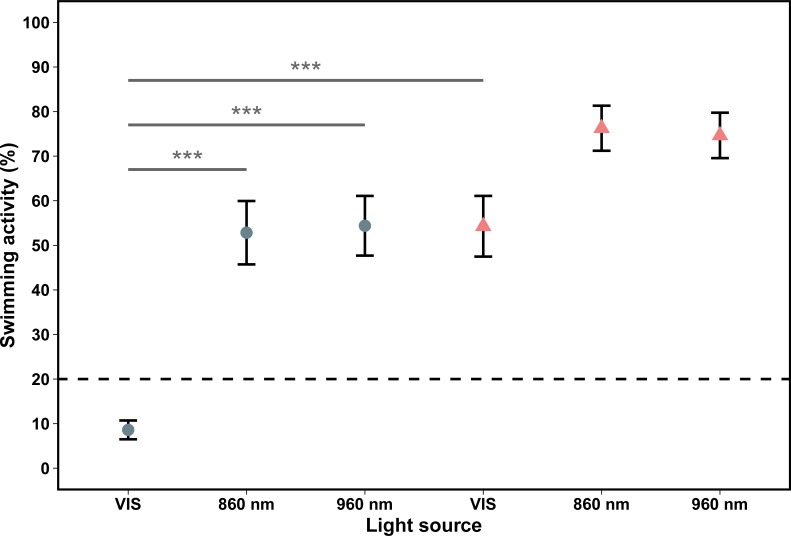
Swimming activity [%] of 96 hpf (blue circles) and 120 hpf (red triangles) zebrafish larvae under different light sources. The x-axis indicates the three tested light sources and the y-axis shows the swimming activity (%) of the zebrafish larvae. Shown are mean ± standard error. Sample size in each experiment: N = 30. ***P < 0.001. (VIS = visible light (blue-white-light, 440–700 nm, 860 nm = NIR light with peak illumination at 860 nm; 960 nm = NIR light with peak illumination at 960 nm).

The swimming activity of 96 hpf zebrafish larvae differed between the experiments with different light sources (Kruskal-Wallis test, χ^2^ = 29.339, P ≤ 0.001, Fig 2). They showed a significantly higher swimming activity under exposure of 860 nm or 960 nm NIR light (52.83 and 54.38%, respectively) than under exposure of VIS light (8.59%) (Wilcoxon rank sum test for unpaired samples, 860 nm: W = 169.5, P ≤ 0.001; 960 nm: W = 102, P ≤ 0.001, Fig 2, left panel). The 120 hpf larvae showed at NIR 860 nm and 960 nm sources a swimming activity of 76.26% and 74.65%, respectively, therefore, the 96 hpf and 120 hpf old larvae did not differ significantly in swimming activity, neither at 860 nm (Wilcoxon rank sum test for unpaired samples, W = 602.5; P = 0.072, Fig 2) nor at 960 nm NIR light (Wilcoxon rank sum test for unpaired samples, W = 318; P = 0.155, Fig 2). Thus, the swimming activity of 120 hpf zebrafish larvae did not differ under the different light sources (Kruskal-Wallis test, χ^2^ = 4.8308, P > 0.089, Fig 2).

Under VIS light, however, the mean swimming activity of 120 hpf zebrafish larvae was 54.27% and thus 6.3 times higher than mean swimming activity of the 96 hpf larvae under the same light condition (Wilcoxon rank sum test for unpaired samples, W = 815; P ≤ 0.001, Fig 2).

### Allocation time under NIR and VIS light

**Fig 3:**
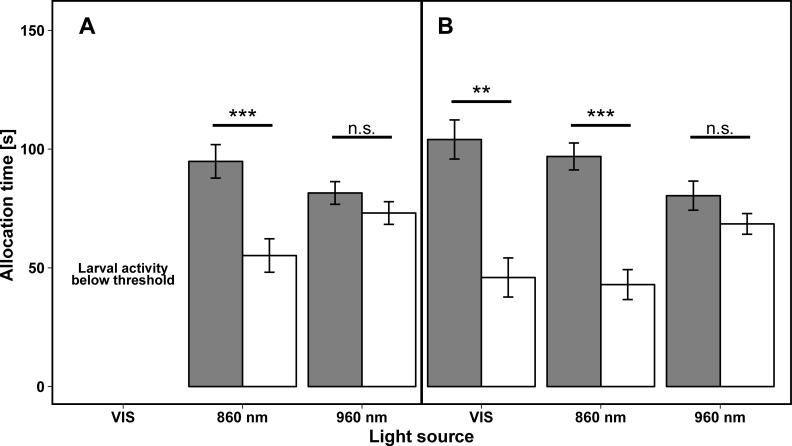
Mean allocation time [s] of both larval stages of zebrafish larvae under different light conditions. Bars present mean allocation time [s] ± standard error (which sum up to 150 s) of zebrafish larvae (A: 96 hpf, B: 120 hpf) in halves of vessels illuminated with NIR light (white bars) and radiated by the thermal control (dark grey bars) (N_96hpf/VIS_ = 3, N_96hpf/860 nm_ = 20, N_96hpf/960 nm_ = 22, N_120hpf /VIS_ = 20, N_120hpf /860 nm_ = 28, N_120hpf /960 nm_ = 28). The x-axis represents the wavelengths of the three different used light sources (VIS (440–700 nm), 860 nm; 960 nm) and asterisks indicate significant differences compared to thermal control (**P < 0.01, ***P < 0.001, n.s. = not significant).

When a 860 nm NIR light source and the thermal source (control) were simultaneously presented opposite to each other, both larval stages spent significantly more time [s] on the control side with the thermal source than on the NIR half (Wilcoxon signed rank test, 96 hpf: V = 120; P ≤ 0.001; 120 hpf: V = 385; P ≤ 0.001, Fig 3A and 3B). In contrast to this, we found no preference for either side in larvae of both larval stages, when exposed to 960 nm NIR light and the thermal source (Wilcoxon signed rank test, 96 hpf: V = 169.5, P = 0.691, 120 hpf: V = 216.5; P = 0.449, Fig 3A and 3B). Under VIS light, the 120 hpf larvae spent significantly more time [s] on the side with the thermal source than on the side exposed with VIS light (Wilcoxon signed rank test, V = 211; P = 0.01, Fig 3B). Due to a swimming activity below the pre-defined threshold we could not analyse allocation time [s] in 96 hpf larvae under VIS light (Fig 3A).

**Fig 4:**
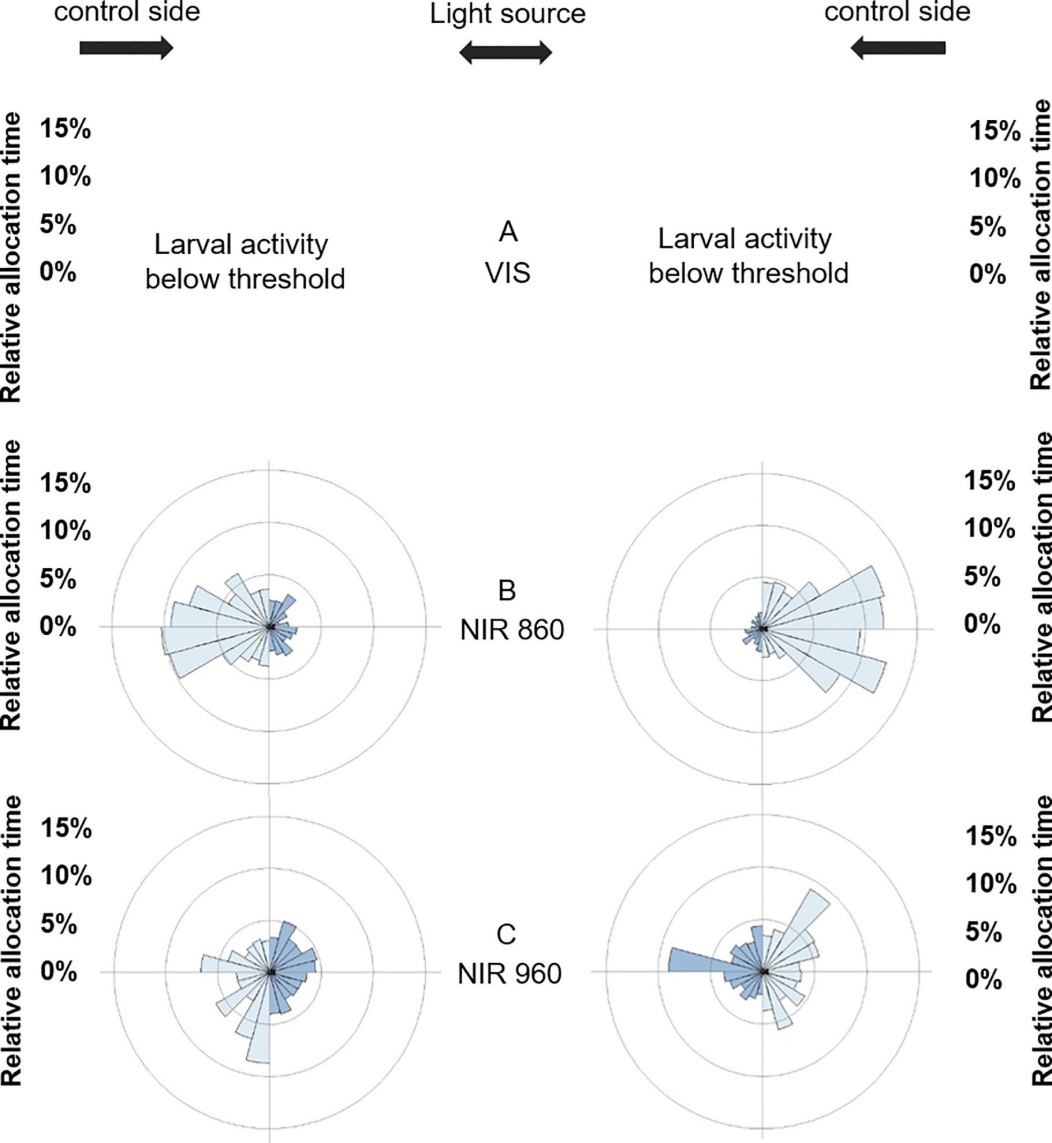
Sector diagram of allocation preference for 96 hpf zebrafish. The blue bars represent the mean allocation time [%] of zebrafish larvae in the test vessel under VIS light (A), NIR light at 860 nm (B) and under NIR light at 960 nm (C). The diagram is divided into 24 sectors, whereby each sector illustrates 15° (Left side: N_96hpf/VIS_ = 2, N_96hpf/860 nm_ = 10, N_96hpf/960 nm_ = 9; Right side: N_96hpf/VIS_ = 1, N_96hpf/860 nm_ = 12, N_96hpf/960 nm_ = 11; except larvae below 20% activity threshold).

**Fig 5:**
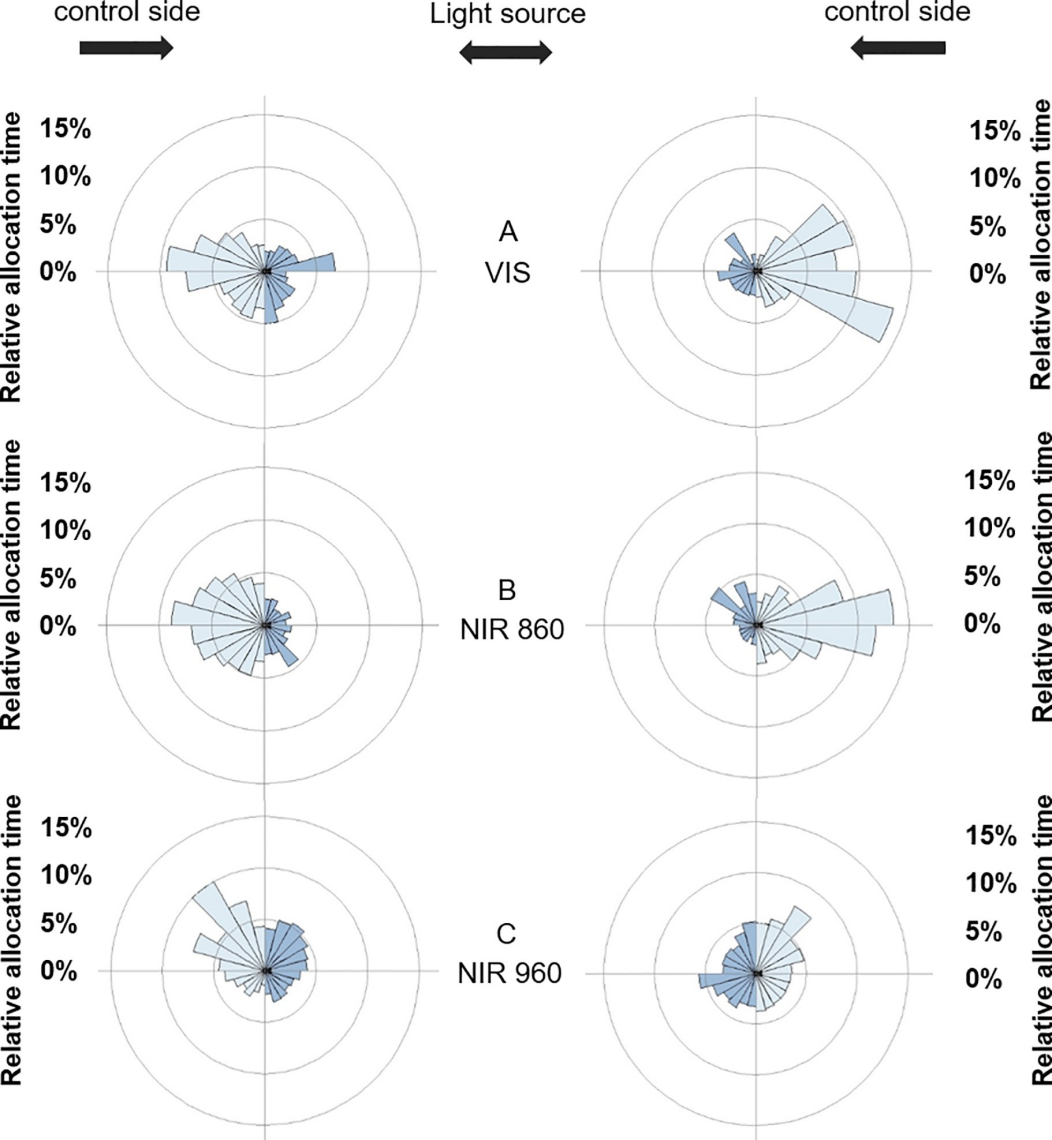
Sector diagram of allocation preference for 120 hpf zebrafish. The blue bars represent the mean allocation time [%] of zebrafish larvae in the test vessel under VIS light (A), NIR light at 860 nm (B) and under NIR light at 960 nm (C). The diagram is divided into 24 sectors, whereby each sector illustrates 15° (Left side: N_120hpf/VIS_ = 10, N_120hpf/860 nm_ = 15, N_120hpf/960 nm_ = 15; Right side: N_120hpf/VIS_ = 10, N_120hpf/860 nm_ = 13, N_120hpf/960 nm_ = 13; except larvae below 20% activity threshold).

When test vessels were divided into 24 sectors of 15 degrees each [36] to visualise the relative allocation time in the test vessel per sector, we found a similar pattern (Fig 4). Larvae of both larval stages spent more time on the control side when the light side was illuminated with VIS light and 860 nm NIR light (Figs 4 and 5). Larvae of both larval stages did not discriminate between both sides when the 960 nm NIR light source was provided in combination with the thermal source (Figs 4 and 5).

### Head orientation

The 96 hpf and 120 hpf larvae directed their heads significantly more often towards the control side than to the 860 nm NIR light, irrespective of whether the thermal source was presented on the right or on the left side (Table 2; 96 hpf: *R* = 0.75, P < 0.001 (left), *R* = 0.78, P < 0.001 (right); 120 hpf: *R* = 0.53, P < 0.01 (left), *R* = 0.81, P < 0.001 (right)). Surprisingly, under NIR at 960 nm, the 96 hpf larvae showed also a clear preferred head position, since the head of the larvae was pointed significantly more often to the control side (Table 2; *R* = 0.67, P < 0.01 (left), *R* = 0.55, P < 0.05 (right)) whereas the head position of 120 hpf larvae did not point more often to the control side (Table 2; *R* = 0.29, P = 0.26 (left), *R* = 0.37, P = 0.12 (right)). Under VIS light head orientation of 120 hpf larvae pointed significantly more often to the control side when it was on the right but not when it was on the left (Table 2; *R* = 0.32, P = 0.35 (left); *R* = 0.53, P < 0.05 (right)). The analysis of the mean angle [°] did not provide additional information and is listed in supplemental material (S1 Table).

**Table 2:** Length of the mean directional vector *R*. Missing data result from activity level below threshold (for sample size see Figs 4 and 5).

light spectrum	Exposure from the left side		Exposure from the right side	
	96 hpf	120 hpf	96 hpf	120 hpf
VIS	-	0.32 ^n.s^	-	0.53 *
NIR 860 nm	0.75 ***	0.53 **	0.78 ***	0.81 ***
NIR 960 nm	0.67 **	0.29 ^n.s.^	0.55 *	0.37 ^n.s^

*P < 0.05

**P < 0.01

***P < 0.001

n.s. = not significant.

### Temperature of light and thermal sources

The thermal source had a mean temperature of 30.36 ± 0.43°C, the VIS light source had a mean temperature of 30.58 ± 0.45°C, and the 960 nm NIR light source had a mean temperature of 30.82 ± 0.44°C (Table 3). The temperature of the 860 nm NIR light source was on average 33.28 ± 0.18°C. The temperature of the thermal source differed from the one of the VIS light source by 0.22°C, and from the NIR 960 nm light source by 0.46°C. The difference between the 860 nm NIR and the thermal source was on average 2.92°C.

**Table 3:** Mean temperature [°C] ± standard deviation of the used light sources (N = 30) and the temperature differences between the thermal source and the light source.

light spectrum	Mean temperature [°C] after the onset of 5 min (N = 30)	Difference between the thermal source and the corresponding light source [°C]
VIS	30.58 ± 0.45	0.22
NIR 860 nm	33.28 ± 0.18	2.92
NIR 960 nm	30.82 ± 0.44	0.46
Thermal source	30.36 ± 0.43	-

The temperature measured at 5 positions inside the vessel did not differ neither under VIS (440–700 nm), NIR 860 nm, NIR 960 nm light source nor under the thermal source (S2 Table).

## Discussion

We investigated whether zebrafish larvae of two different larval stages (96 hpf and 120 hpf) were sensitive to NIR light at a peak illumination of 860 nm or 960 nm, and to VIS light (440–700 nm), respectively. Regarding swimming activity (I), allocation time (II), relative allocation time (III), head orientation (IV), and length of the mean directional vector *R* (V), our results demonstrate that zebrafish larvae of both larval stages showed a clear negative phototactic response towards NIR light with a peak illumination at 860 nm and VIS light, but not to NIR light at 960 nm wavelength. Therefore, we conclude that zebrafish larvae are able to sense NIR light at 860 nm wavelength. This is the first time that a solid study was performed to discriminate the perception towards different NIR wavelengths in zebrafish larvae of two different larval stages. Thus, our findings are crucial to all experiments using zebrafish larvae in standard testing procedures under NIR light conditions because most experimental devices use 850 nm light sources to provide a presumed dark environment [24].

In general, the response pattern to light conditions depends on the age of zebrafish larvae [7, 30, 31]. Younger larvae (96 hpf) are less active than older larvae (>120 hpf) and the mean resting time decreased with increasing age [30, 31]. We found similar results in our experiments. The 120 hpf larvae showed a higher swimming activity under VIS light than the 96 hpf larvae (54.27% versus 8.59%). Due to the fact that the observed swimming activity in our study is similar to those found in previous studies [7, 30, 31], the results based on our custom-built computer vision system were comparable to commercially available systems used in other studies.

Since all light sources emitted radiation, we simultaneously provided a non-illuminated thermal source to control for a possible effect of temperature on the swimming activity. The heat distribution within the test vessel due to emitted radiation of the thermal source did not differ from heat distribution due to radiation emitted by the VIS (440–700 nm), NIR 860 nm and NIR 960 nm light source (S2 Table). No thermal gradient was detectable within the petri dishes based on the small size of the test vessel (35 mm diameter) and the distance to the light sources (10.5 cm) (S2 Table). Thus, thermal radiation or a thermal gradient could not explain the observed behavioural differences in zebrafish larvae in our tests [36, 39]. Similar to previous studies that exclude thermotaxis as an explaining factor, there are indications that our allocation preferences were not due to thermal radiation but were based on the perception of NIR light possibly due to photoreceptors [36, 39].

The hypothesis that zebrafish larvae are not sensitive to NIR light is based on the study with zebrafish larvae of 120–168 hpf by Brockerhoff et al (1995) [29]. They detected no eye movement (optomotor response; OMR) of larvae fixed with a needle to the petri dish in response to illuminated rotating stripes (750 nm NIR light). Optomotor experiments using fixed fish, therefore, do not allow for properly analysing visual perception in fish. In our study, we used a state-of-the-art experimental set-up, which was designed to detect the NIR sensitivity of fish species under controlled conditions [39]. Obviously, the set-up of the experiment plays an important role regarding visible sensitivity of light conditions as shown by the following studies: Kobayashi et al. (2002) [44] found no reaction to NIR over 800 nm by studying the OMR of two strains of Nile tilapia (*Oreochromis niloticus*). When the cardiac-conditioning technique was used, in which the fish learned to associate a NIR or green light stimulus (conditioning stimulus) with a mild electric shock (unconditioning stimulus), however, the same species showed a visual sensitivity to NIR over 850 nm [34]. Moreover, Shcherbakov et al. (2012) [39] demonstrated the sensation of NIR light at a spectral range of 850 nm—950 nm in Nile tilapia by using an appropriate behavioural experiment.

Adult zebrafish (6 months old) exhibit a positive phototaxis towards light sources with a maximum wavelength in the range of 845–910 nm NIR light [36]. They spent 3.4 times more time in the half illuminated with NIR of 825–890 nm than in the non-illuminated control side. The same was true when adult zebrafish could choose between exposure to VIS light or no light. It is known that adult zebrafish show a different reaction to light than zebrafish larvae [45]. These results are supported by our findings, because in contrast to adult zebrafish, zebrafish larvae exhibited a negative phototactic behaviour towards the side illuminated with NIR light at 860 nm wavelength. Shcherbakov et al. (2013) [36] defined a threshold for NIR sensitivity in adult zebrafish of wavelengths up to 910 nm, as the exposure to longer wavelengths resulted in no behavioural response. Thus, adult zebrafish are able to react to NIR light of a range of 825 nm—910 nm. We found similar findings in zebrafish larvae at 96 hpf and 120 hpf. They did not show a behavioural reaction to NIR 960 nm. The lack of response might be due to an underrepresentation of photoreceptors with a sensitivity to wavelengths longer than 910 nm [36]. To investigate if the reaction is retina related or whether non-visual photoreceptors are involved, further experiments have to be conducted e.g. with blind or eyeless fish. Fernandes et al. 2012 [46] showed that eyeless zebrafish larvae swam towards a light stimulus as light perception was mediated by deep brain photoreceptors. The authors identified neurons of the preoptic region of the hypothalamus as photoreceptors for dark photokinesis [46]. Such findings are very important for the current study, since the mechanism for the sensitivity of NIR perception could not be clarified with our experimental set-up. The NIR perception in zebrafish larvae might be an adaptation to the characteristics of the natural preferred habitat [35, 36]. Fish species living in highly transparent aquatic habitats often show a low sensitivity to NIR [36]. In clear water, NIR light over 930 nm only passes through the water surface by a few cm, however, NIR light (between 806–847 nm) passes through water up to 2 m [36]. *D*. *rerio* lives in shallow waterbodies around the Ganges and Brahmaputra river basins in north-eastern India, Nepal and Bangladesh [47, 48]. In their natural habitats, they are found in relatively clear waters with a depth up to 103 cm and a transparency higher than 15 cm [47, 49, 50]. The preferences for this kind of water quality is correlated to a low NIR spectral sensitivity [36]. As zebrafish inhabit slow moving and shallow waters, NIR wavelengths are presented in the natural habitat of this species and may have shaped the sensitivity of their visual system [36, 47]. The optical properties in the natural water habitat of zebrafish seem to correlate with the visual pigments and photo pigment spectral sensitivity in this fish species [51, 52]. Based on the ecological adaptation zebrafish have evolved in their natural habitats, it is not surprising that our study confirms that 96 hpf and 120 hpf zebrafish larvae with are sensitive to NIR light at 860 nm.

## Conclusion

Opposite to previous knowledge, our results provide evidence that 96 hpf and 120 hpf zebrafish larvae are sensitive to light of 860 nm wavelength within the NIR spectrum. They exhibited a clear negative phototaxis to an 860 nm light source and to VIS light. Our study is highly relevant to all studies using zebrafish larvae as test organism, because most of these studies are by default performed under NIR at 850 nm to mimic a dark environment. Thus, previous results should be re-interpreted due to the negative phototactic response in zebrafish larvae under NIR at 860 nm.

## Supporting information

S1 Fig Profiles of the spectrum of the used VIS, NIR 860 nm and NIR 960 nm light sources and thermal source. (EPS)

S2 Fig Graphic illustration of the determination of the position of the larva in the test vessel. (TIF)

S1 Table Mean angle [°] of the fish head position regarding the side of the exposure. (DOCX)

S2 Table Water temperature profiles at different positions within the test vessel (35 mm in diameter) 5 minutes after the onset of the respective light source. (DOCX)
