# Supplementary figures and images for: Zebrafish larvae show negative phototaxis to near-infrared light
Source: PLoS One. 2018 Nov 28;13(11):e0207264. doi: 10.1371/journal.pone.0207264 (PMC6261574; doi:10.1371/journal.pone.0207264)

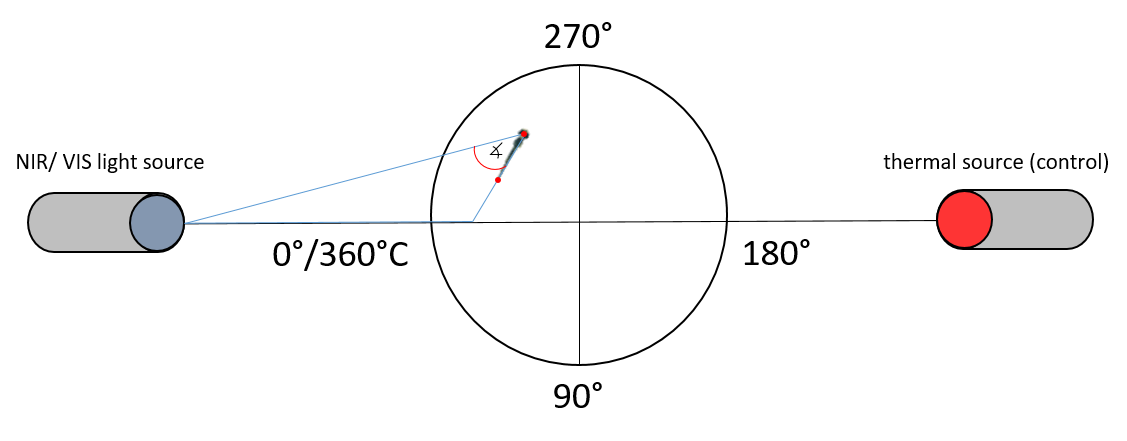

Supplement: S2 Fig — (TIF) [file pone.0207264.s002.tif]
