# Supplementary material for: Zebrafish larvae show negative phototaxis to near-infrared light
Source: PLoS One. 2018 Nov 28;13(11):e0207264. doi: 10.1371/journal.pone.0207264 (PMC6261574; doi:10.1371/journal.pone.0207264)
Supplement: S1 Table — (DOCX) [file pone.0207264.s003.docx]

S1 Table. Mean angle [°] of the fish head position regarding the side of the exposure.

| light spectrum | Exposure from the left side | | Exposure from the right side | |
| --- | --- | --- | --- | --- |
|  | **96 hpf** | **120 hpf** | **96 hpf** | **120 hpf** |
| VIS | - | 206.33 ± 121.13 | - | 206.20 ± 130.56 |
| IR 860 nm | 167.50 ± 45.76 | 177.60 ± 146.67 | 185.17± 41.96 | 153.68 ± 160.63 |
| IR 960 nm | 174.11 ± 60.39 | 176.09 ± 124.52 | 152.67 ± 64.35 | 179.83 ± 132.36 |
