# Supplementary material for: Zebrafish larvae show negative phototaxis to near-infrared light
Source: PLoS One. 2018 Nov 28;13(11):e0207264. doi: 10.1371/journal.pone.0207264 (PMC6261574; doi:10.1371/journal.pone.0207264)
Supplement: S2 Table — (DOCX) [file pone.0207264.s004.docx]

**S2 Table: Water temperature profiles at different positions within the test vessel (35 mm in diameter) 5 minutes after the onset of the respective light source and thermal source.** Temperature was measured in the right and left sides, in the centre and at the top and the bottom of the test vessel (5 times exposed from right, 5 times from left; N = 5)

| Light source | exposed side | positions within the test vessel (mean + sd) | | | | |
| --- | --- | --- | --- | --- | --- | --- |
|  |  | left | center | right | top | bottom |
| VIS | right | 26.14 ± 0.14 | 26.30 ± 0.25 | 26.40 ± 0.15 | 26.42 ± 0.16 | 26.46 ± 0.16 |
|  | left | 26.38 ± 0.16 | 26.06 ± 0.19 | 26.06 ± 0.11 | 25.98 ± 0.08 | 26.06 ± 0.11 |
| IR 860 nm | right | 26.16 ± 0.11 | 26.14 ± 0.11 | 26.18 ± 0.08 | 26.12 ± 0.08 | 26.06 ± 0.08 |
|  | left | 26.28 ± 0.08 | 26.26 ± 0.13 | 26.12 ± 0.10 | 26.16 ± 0.12 | 26.18 ± 0.10 |
| IR 960 nm | right | 26.28 ± 0.09 | 26.24 ± 0.11 | 26.30 ± 0.18 | 26.32 ± 0.14 | 26.30 ± 0.12 |
|  | left | 26.30 ± 0.11 | 26.26 ± 0.23 | 26.22 ± 0.16 | 26.22 ± 0.13 | 26.20 ± 0.18 |
| thermal source  (IR 860 nm + filter) | right | 26.14 ± 0.11 | 26.12 ± 0.13 | 26.08 ± 0.08 | 26.10 ± 0.07 | 26.04 ± 0.15 |
|  | left | 26.24 ± 0.09 | 26.18 ± 0.13 | 26.14 ± 0.08 | 26.10 ± 0.00 | 26.12 ± 0.08 |
